# Digital (Two-finger) versus Video Laryngoscopy for Nasogastric Tube Insertion in Intubated Patients; a Clinical Trial Study

**DOI:** 10.22037/aaem.v9i1.1281

**Published:** 2021-08-16

**Authors:** Mehdi Nasr Isfahani, Elahe Nasri Nasrabadi

**Affiliations:** 1 Emergency Medicine Department, Alzahra Hospital, Isfahan University of Medical Sciences, Isfahan, Iran.

**Keywords:** Laryngoscopy, Enteral Nutrition, Laryngoscopy, Intubation, Gastrointestinal

## Abstract

**Introduction:**

Performing Nasogastric Tube (NGT) insertion is very challenging in anesthetized and intubated patients. The current study aimed at comparing Digital (two-finger) and Video Laryngoscopy methods for NGT insertion in the mentioned patients.

**Methods:**

The present single-blind clinical trial was performed on 76 intubated patients, who were randomly divided into two groups. Groups A and B underwent Video Laryngoscopy and Digital (two-finger) methods, respectively. Then, the success rate, the number of attempts to insert NGT, duration of insertion, hemodynamic parameters, and patients’ satisfaction level were recorded and compared between groups.

**Results:**

The mean duration of NGT insertion in group A was significantly higher than that of group B (19.07 ± 2.07 vs 11.53 ± 2.16 seconds; P value=0.001). The success rate was higher in group B (94.7% vs. 78.9%; P value=0.042). Considering the interfering factors such as patients’ body mass index (BMI), the odds of success in group B was reported to be 8.49 times higher than that of group A (P value =0.028).

**Conclusion:**

Digital method can be considered as a safe and appropriate method of NGT insertion for intubated cases with high success rate and speed of performance.

## 1. Introduction:

 One of the most frequent procedures in emergency departments (ED) is Nasogastric Tube (NGT) insertion. It is applied in mechanically ventilated patients for different purposes, such as decompression of stomach, reducing the pressure on the lungs for better breathing and respiration, preventing the risks of emptying the gastric contents during aspiration, and feeding (1-4). Although this procedure is frequently done, it is very challenging to insert NGT in anesthetized patients due to patients’ lack of cooperation and inability to swallow. In addition, in many cases, attempts to insert the NGT result in failure to properly insert the tube into the stomach (5).

There is a variety of NGT insertion methods for intubated patients in EDs, among which Digital and Video Laryngoscopy are two routine methods that can be employed (6). The Digital method is a practical method that is performed in most EDs, in which NGT insertion is performed with the help of the fingers by depressing the tongue and creating a suitable space (7). In video Laryngoscopy, which is an alternative to conventional methods that are usually performed blindly, a color monitor that directly shows the glottis is used (8). The present study was performed to evaluate the success rates of Digital (Two-finger) and Video Laryngoscopy methods in insertion of NG tube in intubated patients in the EDs.

## 2. Methods:

***2.1. Study design and setting***

The present study was a single-blind randomized clinical trial (number of RCT: NCT04414839), which was conducted on intubated patients in the EDs of Al-Zahra and Ayatollah Kashani Hospitals in Isfahan, Iran during 2018-2019. The study protocol was approved by Ethics Committee of Isfahan University of Medical Sciences (under the number: IR.MUI.MED.REC.1397.287) and a written informed consent was obtained from the participants before inclusion in the study.

***2.2. Participants***

18 - 65-year-old patients in the mentioned EDs, who underwent ***rapid sequence intubation*** (RSI) and required NGT insertion were included. Cases with skull base fracture symptoms, coagulopathy and hemorrhagic disorders, ***maxillofacial traumas*** leading to the deformity and disturbance in NGT insertion, diseases and anomalies of the upper respiratory tract, deviated nasal septum, ***nostril stenosis,*** esophageal disorders (esophageal stricture, esophageal varices), and a history of head and neck radiotherapy, as well as patients intubated in and transferred from other centers were excluded. In addition, patients with more than two unsuccessful attempts at NGT insertion were excluded from the study.

***2.3. Data gathering***

Patients were randomly divided into two groups using random allocation software and simple randomization method. Each group was subjected to NGT insertion using either the Digital or the Video Laryngoscopy method. First, demographic data including patients’ age, sex, weight, and height were recorded. Then, all patients underwent continuous blood pressure (BP) monitoring. Then, their pulse rate (PR), systolic blood pressure (SBP), diastolic blood pressure (DBP), and oxygen saturation percentage (O2sat) were recorded at baseline.

In addition, the possible adverse events including mucosal bleeding, hemodynamic abnormalities (hypertension, tachycardia, and arrhythmia), esophageal perforation, and kinking and twisting of the NGT were recorded for all patients. PR, SBP, DBP, and O2sat were also evaluated and recorded at the end of the procedure. 

Moreover, inserter's satisfaction level was scored and recorded on a scale ranging from 1 (low satisfaction) to 10 (very high satisfaction).

***2.4. Procedure***

After making sure that the patient’s condition was favorable, and the intubation and resuscitation devices were ready, the patients were oxygenated with Ambu bag and mask for three minutes. Then, for induction of analgesia and anesthesia, all patients received the same medicines as follows: Fentanyl 3 μg/kg, Etomidate 0.3 mg/kg, and Succinylcholine 1.5 mg/kg). Then, they underwent intubation using RSI technique with tubes of similar size (Men: 7.5-8mm internal diameter and Women: 7-7.5 mm internal diameter). Moreover, for all patients, endotracheal tube (ETT) cuff was also inflated with air to maintain the ETT cuff pressure within the range of 15-25 cm of water and the cuff was then fixed in its proper place. Whether NGT insertion was successful or not, we didn’t open the cuff for better insertion and cuff pressure was maintained.

Before NGT insertion, the appropriate ***length of NGT for proper insertion*** into the patient’s stomach was first measured by placing the NGT tip on the patient’s xiphoid and extending it to the tip of the nose and then to the patient’s earlobe.

It must be mentioned that since the patients could not cooperate to perform deep exhalation through the nose, the larger nostril was selected for NGT insertion based on the size of nostrils.

All patients received 3 mL of ***lubricant*** gel and ***lidocaine anesthetic*** along with phenylephrine nasal drops in their selected nostril 5 minutes before NGT insertion. Moreover, NGT No. 14-16 was used for all patients in accordance with the selected method.

In the NGT Video Laryngoscopy group (group A), first, the GlideScope blade was inserted under direct vision via the color monitor through the patient’s mouth by employing jaw-thrust maneuver to preserve the cervical spine and by raising the tongue to obtain a better view of the larynx space. Then, NGT was inserted through the selected nostril, advanced through the esophagus under direct vision to meet the measured length, and was fixed after confirmation.

**Figure 1:**
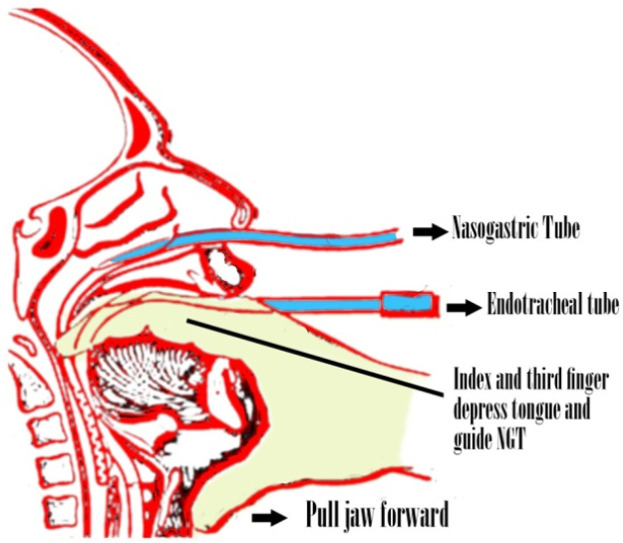
Digital (two-finger) method in supporting nasogastric tube (NGT) insertion for intubated patients (From Samuels LE. Nasogastric and feeding tube placement. In: Roberts JR, Hedges JR, editors. Clinical procedures in emergency medicine. 4th ed. Philadelphia: Saunders; 2004. pp. 794-816)

In the NGT Digital Intubation group (group B), the second and third fingers were placed in the posterior pharynx and the tongue was depressed downwards. The NGT was passed through the nose into the posterior pharynx with the fingers in the pharynx to reach the esophagus. The thumb was placed under the jaw and pushed it forward to pave the way for tube insertion (Fig. 1).

It should be noted that in both groups NGT was inserted by a senior emergency resident, who had proven competency in NGT insertion for anesthetized intubated patients using both techniques. 

In addition, to check the proper placement of the NGT, an irrigation syringe instilled a 30cc air bolus into the patient's stomach and stethoscope was used to simultaneously listen over the epigastrium. If the entrance of air into the stomach produced a whooshing sound, the proper placement of NGT was confirmed.

**Figure 2:**
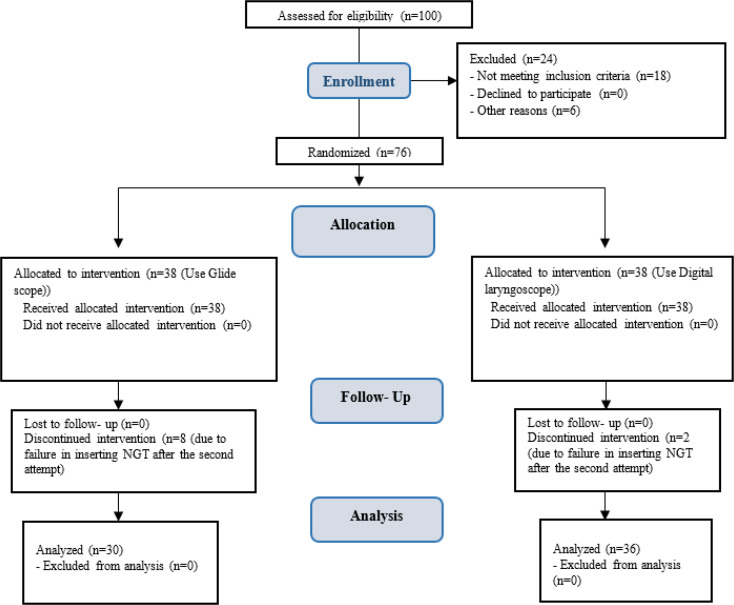
Study flow chart

If the first attempt at NGT insertion was successful, it was considered as a successful attempt. However, if the first attempt failed, the NGT was completely removed and after cleaning was inserted again using the same method. If both NGT insertion attempts were unsuccessful, the NGT was inserted using another method (Fig. 2).

In all groups, the duration of NGT insertion was measured by another individual using a stopwatch. The start time was when NGT entered the selected nostril, and the end time was when the measured NGT length had fully entered the stomach.

It must be mentioned that blinding of the researcher was not possible due to the different natures of the two methods of NGT insertion; however, the data recorder and analyst were not informed of the difference of the two methods. Therefore, the present study was a single-blind study.

***2.5. Statistical analysis***

Considering a confidence interval of 95%, a test power of 80%, and based on the findings of previous studies, reporting the success rates of 54% and 92% for successful NGT insertion in the conventional and Digital methods, respectively, the sample size of 38 patients was considered for each group. The mentioned sample was selected using convenience random sampling. Finally, the collected data were entered into SPSS software (ver.23). The data were presented as mean±standard deviation or frequency (percentage). In addition, based on the results of Kolmogorov-Smirnov test indicating the normality of data distribution, an independent samples *t*-test and Fisher’s exact test were used to compare the means of quantitative variables and to compare the frequency distribution of discrete data, respectively. Moreover, logistic regression with the enter method was used to evaluate the factors affecting the odds of success in NGT insertion. In all analyses, significance level was considered to be p values less than 0.05.

## 3. Results:

 Group A included 22 (57.9%) males and 16 (42.1%) females with the mean age of 51.05 ± 10.14 years. Group B consisted of 17 (44.7%) males and 21 (55.3%) females (p = 0.359) with the mean age of 53.39±10.53 years (p = 0.327). There was no significant difference between the two groups in terms of height (p=0.106), weight (p=0.330), and BMI (p=0.836). 

In addition, the success rate of 94.7% recorded for group B was significantly higher than the success rate of 78.9% obtained for group A (p=0.042). In addition, in more than 80% of patients in group B the first insertion attempt was successful (p= 0.043). The mean duration of insertion in group B with 11.53 ± 2.16 seconds was significantly lower than that of group A with 19.07 ± 2.07 seconds (p= 0.001) (Table 1).

Furthermore, evaluation of the mean of each of the hemodynamic parameters including PR, SBP, DBP, and O2sat indicated that there was no significant difference between the two groups before and after NGT insertion (P-value> 0.05) (Table 2).

Furthermore, inserter's satisfaction level in group A with the mean score of 6.66±2.82 was significantly lower than that of group B with the mean score of 8.82 ± 1.99 (p=0.001). In addition, the only adverse event of this procedure was kinking and twisting of the NGT in groups A and B with 18.4% and 7.9%, respectively, the rate of which was not significantly different between the two groups (p=0.175).

The results of evaluating the factors affecting the success of NGT insertion indicated that the success rate was significantly higher in group B compared with group A (Odds: 8.49; 95% CI: 1.26-27.12; p = 0.028). In addition, with increase in BMI, the odds of success in NGT insertion decreased 0.80 times (p= 0.013); however, age (p =0.725) and sex (p = 0.763) had no significant effect on the success rate of NGT insertion (Table 3).

## 4. Discussion:

Based the results of the present study, the Digital Intubation method was more successful than the Video Laryngoscopy method (success rate: 94.7 vs. 78.9%). In addition, the frequency of the first successful insertion attempt was significantly higher in Group B compared with Group A. One possible reason for the difference in the success rates in the first attempt may be that using fingers helps open the throat. Many other factors such as choosing the right size, flexibility, and temperature of NGT should be taken into account in the process of insertion. For instance, choosing a larger size would result in less flexibility. 

NGT insertion in ice leads to a higher rate of success in the first insertion attempt (6, 9, 10).

The number of failed attempts using the Digital method is much lower than that of the conventional method. In addition, the speed of providing services when using the Digital method is significantly higher than that of the blind methods. The extent of the patient’s mouth opening, the presence or absence of teeth, the length of the neck, the lack of mechanical complications in the throat and respiratory tract, the experience and skill of the inserter, and the length of the physician’s fingers can be regarded as various factors that affect the quality of performing and success rate of the Digital method (7). On the other hand, Video Laryngoscopy reduces not only the failure rate for the first attempt but also the side effects on the soft tissue of the throat compared with the conventional method. This method prevents the formation of pneumonia caused by the entry of gastric contents into the lungs and increases the accuracy of directing NGT to larynx (8). Despite all the above-mentioned positive points about this method, the disadvantages of this method are also significant. Some drawbacks of this method include the use of stylet to adjust the insertion tube, the hardship of entering the tube through the glottis, and the limitations of utilizing the device due to the limited mouth opening. In addition, some devices employed in this method are sometimes heavy, while lighter and more portable devices have a smaller screen (8).

Furthermore, the present study revealed that the duration of NGT insertion in group B was significantly lower than that of group A, and the emergency physician performed the NGT insertion process at very high speed in group B, which naturally led to inserter's higher satisfaction (11.5 vs. 19.7 seconds). Finding the epiglottis employing the Video Laryngoscopy method increased the time of insertion, while using fingers and depressing the tongue with two fingers in the Digital method led to better direction of the tube to the esophagus, which minimizes the possibility of tube twisting and physical damage.

Tantri’ et al.’s study revealed that the success rate of NGT insertion at the first attempt in the finger method was significantly higher than that of the reverse Sellick maneuver method. In addition, the incidence of complications in the finger method has been very low. Therefore, they stated that the finger method is a feasible and safe method for NGT insertion (11). Although the finger method used in their study was different from the finger method employed in the current study, it can be stated that despite not using special and advanced devices and lack of changes in the position of the patient’s head and neck, this method could yield higher success rate by taking advantage of the knowledge of the patient’s anatomical condition.

The hemodynamic parameters of patients before and after NGT insertion revealed that patients had stable clinical symptoms, and PR, SBP, DBP, and O2sat did not significantly change in any of the patients. However, in their article about cardiovascular responses to the NGT insertion, Fassoulaki et al. found that SBP significantly increased immediately after NGT insertion using both blind finger and laryngoscope methods. However, 3 minutes after the insertion, SBP was still significantly higher only in laryngoscope method. PR had also increased in both methods. O2sat also increased dramatically after NGT insertion. In the end, they concluded that insertion of NGT in anaesthetized patients should ideally be done using the blind finger method (12). The mentioned lack of changes in both methods, despite the presence of advanced devices in the Video Laryngoscopy method, indicates the safety of the Digital method and the ease of using this method. However, the consensus of emergency physicians recommends the use of Video Laryngoscopy method for patients with pharyngeal complications, obese patients, patients with esophageal and upper respiratory tract cancers, or patients with cervical spinal cord injuries.

In addition, the level of inserter's satisfaction in method B (Digital) was reported to be higher than that of method A (Video Laryngoscopy). The mentioned finding can be of great value as consideration of the inserter's satisfaction and their lower annoyance are of particular significance for physicians in clinical interventions. This satisfaction may be attributed to the shorter duration of tube insertion in the Digital method compared with the Video Laryngoscopy method or may be ascribed to the high success rate of tube insertion in the first attempt in Group B. 

Given the lack of differences in tube insertion problems such as NGT kinking and twisting between the two methods, it can be concluded that the Digital method can be used safely and quickly for tube insertion in the EDs. Nevertheless, Tantri et al. found that finger method had a lower blood spot complication and kinking or coiling of NGT, which is possibly due to better guidance of the tube using fingers by fixing the tip of NGT right at the entrance of the esophagus (11).

The results of logistic regression analysis in identifying the factors influencing the successful insertion of NGT revealed that although age and sex did not affect the success rate, the patients’ BMI had a significant effect on the success rate of NGT insertion. The higher the BMI of the patients, the less likely they are to have successful NGT insertion. In addition to this interfering effect, method B increases the success rate many times compared with method A. In a review article by Liao et al., the issue of obesity and its negative effects on tube insertion has been discussed. They noted that tube insertion in obese patients is a major challenge for the emergency physicians, for whom guiding NGT in the right position is difficult due to abnormal anatomy of the glottis, e.g. narrowing of the upper airways, which leads to a significant decrease in SPO2 or functional residual capacity during intubation (13). Since the time-consuming and difficult nature of NGT insertion in obese patients leads to hypoxia, many physicians recommend the use of Video Laryngoscopy method in these patients (13). However, few reports stated that BMI was not a factor influencing the success rate of this process (14). Prominent studies have only focused on airway tracheal tube methods and have not directly mentioned NGT insertion. We suggest that larger studies can address the impact of BMI on the success rate of NGT insertion and its related complications.

Therefore, the main advantage of the present study can be selection of the appropriate method considering the interfering factors such as patients’ BMI, which can cause the least damage to the cervical spinal cord and also have the lowest risk for the patient. In fact, this study revealed that although both methods are common and documented, the use of Digital method can be preferred over the Video Laryngoscopy method due to not applying sophisticated devices, uncomplicated process of learning and training, and its ease of use. On the other hand, the Video Laryngoscopy method can cause fewer physical complications for the patient as it provides better vision for novice residents. The mentioned advantages can be obtained depending on the presence or absence of advanced devices. Although the Video Laryngoscopy method can be used in large and well-equipped hospitals, the Digital method can be used easily and without high costs in small hospitals or clinics, where NGT insertion must be performed as soon as possible.

Since our study demonstrated the lower success rate of the Video Laryngoscopy method, use of Mcgill forceps is recommended for better guiding of tube into the glottis in this method. Also, due to moral issues regarding ICU patients, for whom we are not allowed to insert NGT in the nostrils, it is suggested to insert the NGT directly through the mouth. In addition, from our experience, use of frozen NGT leads to better insertion outcomes. Furthermore, higher BMI level was one of the factors affecting the success rate of NGT insertion. Thus, recruiting those with high BMIs in a larger study to evaluate the exact role of BMI on the success rate of NGT insertion in both Video Laryngoscope and Digital methods is recommended. Digital method can be used in medical centers due to its ease of implementation, practicality, availability, and lack of need for advanced and expensive devices. However, application of new methods and integrating them with conventional ones can yield a more desirable outcome and a very high success rate. It is suggested to conduct future studies with a larger sample size and a similar design in order to achieve more definitive results generalizable to the population.

**Table 1:** Comparing the baseline characteristics of patients between Video Laryngoscopy (group A) and Digital Intubation (group B) groups

**Variables**	**Group A (n=38)**	**Group B (n=38)**	**P-value**
**Gender; male**	22(57.9)	17(44.7)	0.359
**Age; year**	51.05±10.14	53.39±10.53	0.327
**Height; cm**	170.43±9.71	164.92±9.71	0.106
**Weight; kg**	71.51±13.29	67.95±17.82	0.330
**Body mass index**	24.56±3.96	24.79±5.75	0.836
**Success rate**	30(78.9)	36(94.7)	0.042
**1** ^st^ ** attempt insertion**	23(60.5)	31(81.6)	0.043
**2** ^st^ ** attempt insertion**	7(18.4)	5(13.2)	0.744
**Duration of NGT insertion** **; Seconds**	19.07±2.07	11.53±2.16	<0.001

Data are presented as mean ± standard deviation or frequency (%). NGT: nasogastric tube.

**Table 2:** Comparing the hemodynamic parameters before and after nasogastric tube insertion between Video Laryngoscopy (group A) and Digital Intubation (group B) groups

**Variables**		**Group A**	**Group B**	**P-value**
**PR**	**Before**	76.51±6.36	77.91±5.95	0.326
	**After**	78.09±6.25	78.53±6.96	0.772
**SBP**	**Before**	103.37±8.28	103.39±8.46	0.989
	**After**	110.03±7.20	108.00±7.36	0.229
**DBP**	**Before**	66.95±6.41	67.75±7.70	0.624
	**After**	67.92±7.96	68.83±7.96	0.620
**O2sat**	**Before**	97.30±1.61	97.16±1.24	0.670
	**After**	97.91±0.64	97.92±0.67	0.920

Data are presented as mean ± standard deviation**.** PR: Pals rate, SBP: Systolic Blood pressure, DBP: Diastolic Blood pressure, O2sat: Oxygen Saturation.

**Table 3:** Determining the factors affecting the success of nasogastric tube insertion

**Factors**	**Beta**	**S.E.**	**OR (95% CI)**	**P-value**
**Digital vs Video Laryngoscopy**	2.14	0.97	8.49 (1.26-27.12)	0.028^*^
**Age**	0.01	0.04	1.01 (0.94-1.09)	0.725
**Sex**	0.24	0.78	1.27 (0.27-5.85)	0.763
**BMI**	-0.22	0.09	0.80 (0.67-0.95)	0.013^*^

*: P-value less than 0.05 indicates a significant effect on the success rate. BMI: body mass index; OR: odds ratio; CI: confidence interval.

## 5. Limitation:

The small sample size and the impossibility of double-blinding due to the type of devices and the technique of the NGT insertion can be regarded as limitations of the present study. Moreover, the implementation of this study in educational centers by trained assistants can be considered as another limitation. However, it is also worth noting that trained assistants implementing the Digital technique have been able to achieve a high success rate in NGT insertion using fewest readily available devices. The mentioned point can be an indication of the safety of this method.

## 6. Conclusion:

Based on the results of the present study, the success rate of NGT insertion and the number of successful first attempts have been significantly higher in the Digital Method compared with Video Laryngoscopy method. In addition, the speed of performing this process was significantly higher in the Digital method compared with the Video Laryngoscopy method. However, it must be mentioned that the stability of patients’ hemodynamic parameters and the occurrence of adverse events were not significantly different between the two methods. 

## 7. Declarations

### 7.1. Acknowledgements:

We would like to express our gratitude to the staff of Emergency Medicine departments of Al-Zahra and Ayatollah Kashani Hospitals affiliated to Isfahan University of Medical Sciences.

### 7.2. Authors' contribution:

E. NN: Conceptualization; data collection; Investigation; writing the original draft. M. NI.: Conceptualization; methodology; project administration; supervision; review and editing. 

### 7.3. Funding

This work was supported by deputy of research and technology of Isfahan University of Medical Sciences (Grant# 397585).

### 7.4. Conflict of interest:

The authors declare they have no conflict of interest.
